# Cytotoxic 1,3-Thiazole and 1,2,4-Thiadiazole Alkaloids from *Penicillium oxalicum*: Structural Elucidation and Total Synthesis

**DOI:** 10.3390/molecules21030232

**Published:** 2016-02-26

**Authors:** Zheng Yang, Nianyu Huang, Bang Xu, Wenfeng Huang, Tianpeng Xie, Fan Cheng, Kun Zou

**Affiliations:** 1 Hubei Key Laboratory of Natural Products Research and Development, College of Biological and Pharmaceutical Sciences, China Three Gorges University, Yichang 443002, China; yangzctgu@sina.com (Z.Y.); hny115@126.com (N.H.); xubang08@163.com (B.X.); sxdxxtp@foxmail.com (T.X.); 2 China Three Gorges University People’s Hospital & Yichang First People’s Hospital, Yichang 443000, China; 3 Hubei Key Laboratory of Natural Products Research and Development, Medical College, China Three Gorges University, Yichang 443002, China; xyyxy1999@aliyun.com

**Keywords:** *Penicillium oxalicum*, alkaloids, total synthesis, structure elucidation, cytotoxicity

## Abstract

Two new thiazole and thiadiazole alkaloids, penicilliumthiamine A and B (**2** and **3**), were isolated from the culture broth of *Penicillium oxalicum*, a fungus found in *Acrida cinerea*. Their structures were elucidated mainly by spectroscopic analysis, total synthesis and X-ray crystallographic analysis. Biological evaluations indicated that compound **1**, **3a** and **3** exhibit potent cytotoxicity against different cancer cell lines through inhibiting the phosphorylation of AKT/PKB (Ser 473), one of important cancer drugs target.

## 1. Introduction

Numerous natural products with novel structures and distinct biological activities have been discovered as secondary metabolites of insect-derived microbes [1,2]. *Penicillium oxalicum* is one of the most ubiquitous toxigenic fungi found in soil and musty cereal, with many biologically and structurally novel secondary metabolites, such as secalonic acid D [3], oxalicine [4], as well as ergosterol peroxide [5], have been isolated from this fungus.

Heterocyclic compounds are attractive to medicinal chemists because of their unique chemical properties and wide-ranging biological activities. As one of basic five-membered heterocycles, the thiazole substructure is widely found in many bioactive natural products including the cytotoxic compound myxothiazol [6,7,8,9], the sodium channel activator hoiamides A [10,11], and the orally active peptide sanguinamide A [12]. Moreover, the thiadiazole ring has also received increasing attention in recent decades because of its broad-spectrum activities, together with many important therapeutic applications [13,14]. For example, the polycarpathiamines A and B showed significant cytotoxic activity against L5178Y murine lymphoma cells [15], while indole alkaloids containing 1,2,4-thiadiazole rings exhibit anti-viral antiviral activity against the herpes simplex virus 1 (HSV-1) [16].

**Figure 1:**
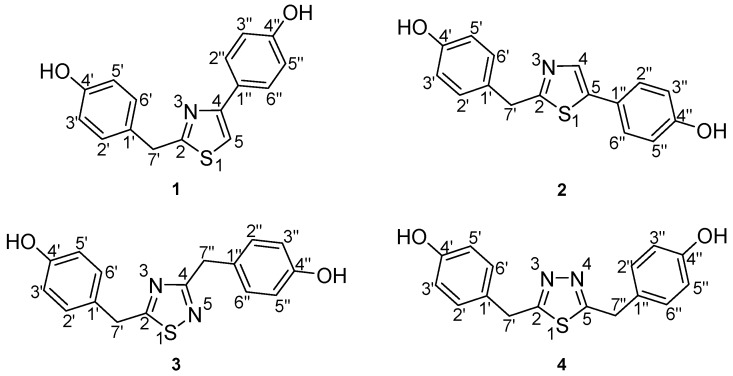
Chemical structures for the two pairs of isomers.

The literature reports different approaches to construct the thiazole and thiadiazole skeleton. In general, the Hantzsch procedure employing thioamides [17,18] or using the condensation and oxidation reaction between cysteine esters and *N*-protected iminoesters [19,20] has become the classic method for the synthesis of thiazoles, and intramolecular or intermolecular cyclization strategies [21,22] are widely used for the preparation of thiadiazoles. In order to determine the final structures for two novel compounds **2** and **3** recently isolated from the secondary metabolites of *Penicillium oxalicum*, two pairs of thiazoles **1** and **2** and thiadiazoles **3** and **4** (Figure 1) were designed and systemically synthesized in this work.

## 2. Results and Discussion

**Figure 2:**
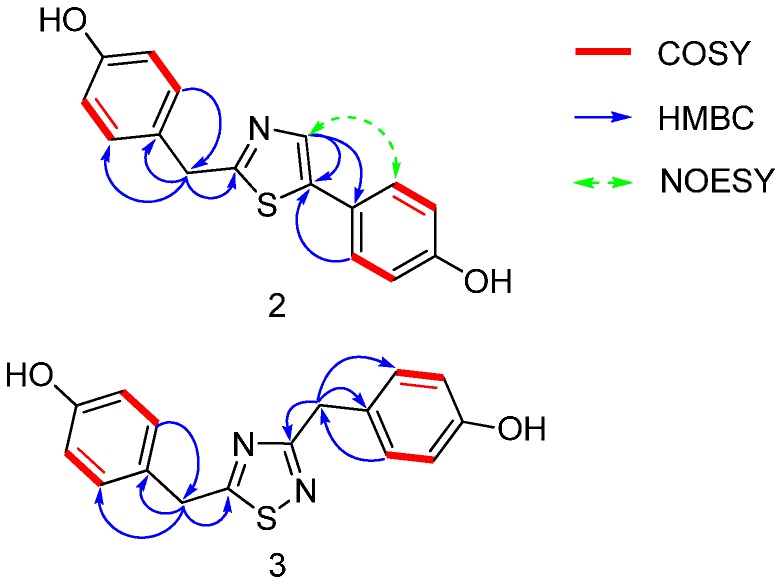
Selected ^1^H-^1^H COSY, HMBC and NOESY correlations of **2** and **3**.

Repeated separation of a 40 L culture extract from *Penicillium oxalicum* using silica gel column chromatography yielded compounds **2** and **3**. Penicilliumthiamine A (**2**) was obtained as a white amorphous powder. Its molecular formula of C_16_H_13_NO_2_S (indicating eleven degrees of unsaturation) was determined by HRESIMS (*m*/*z* 284.0746 [M + H]^+^, calcd. for C_16_H_14_NO_2_S, 284.0740). The ^1^H-NMR spectrum of **2** exhibited two hydroxyl protons (δ_H_ 9.70 and 9.36, which disappeared on exchange with D_2_O), two sets of AA’BB’ spin systems of the *para*-substituted benzene ring at δ_H_ 7.13 (2H, d, *J* = 8.6 Hz, H-2’, 6’), δ_H_ 6.72 (2H, d, *J* = 8.6 Hz, H-3’, 5’) and δ_H_ 7.37 (2H, d, *J* = 8.7 Hz, H-2”, 6”), δ_H_ 6.77 (2H, d, *J* = 8.7 Hz, H-3”, 5”), one olefinic singlet at δ_H_ 7.84 (1H, s, H-4), one benzylic methylene singlet at δ_H_ 4.15 (2H, s, H-7’). The ^13^C-NMR spectrum gave the corresponding resonances. One and two-dimensional NMR techniques (DEPT, ^1^H-^1^H COSY, HSQC and HMBC) permitted assignment of all the ^1^H- and ^13^C-NMR signals for **2** (Table 1, Figure 2). In the HMBC spectrum of **2**, correlations from δ_H_ 9.36 to C-4’ (δ_C_ 156.29), H-2’/6’ (δ_H_ 7.13) to C-1’ (δ_C_ 128.36), C-3’/5’ (δ_C_ 115.38), C-4’ (δ_C_ 156.29), C-7’ (δ_C_ 38.03), and H-3’/5’ (δ_H_ 6.72) to C-1’ (δ_C_ 128.36), C-2’/6’ (δ_C_ 129.99), C-4’ (δ_C_ 156.29), revealed the presence of a 4-hydroxybenzyl group; correlations from δ_H_ 9.70 to C-4” (δ_C_ 157.54), H-2”/6” (δ_H_ 7.37) to C-1” (δ_C_ 121.92), C-3”/5” (δ_C_ 115.89), C-4” (δ_C_ 157.54), and H-3”/5” (δ_H_ 6.77) to C-1” (δ_C_ 121.92), C-2”/6” (δ_C_ 127.63), C-4” (δ_C_ 157.54), revealed the presence of a 4-hydroxybenzene group. Apart from this unit, one olefinic carbon (δ_C_ 136.43), two quaternary carbon δ_C_ (138.76 and 168.66), one sulfur, and one nitrogen atoms remained to be assigned according to the molecular formula. It was clear that eight of eleven degrees of unsaturation came from two phenyl groups, the remaining three degrees of unsaturation had to originate from the -C_3_HNS-moiety, so it had to be a thiazole group. The 4-hydroxybenzyl group attached at the C-2 position was confirmed by the HMBC correlations from H-7’ (δ_H_ 4.15) to C-2 (δ_C_ 168.66). Furthermore, a 4-hydroxybenzene group could be located at C-4 or C-5, which was deduced by the HMBC correlations from H-4 (δ_H_ 7.84) to C-5 (δ_C_ 138.76), C-1’ (δ_C_ 128.36), together with the cross-peaks between H-4 (δ_H_ 7.84) with H-2”/6” (δ_H_ 7.37) in the NOESY spectrum of **2**. However, it was difficult to assign the positions of the 4-hydroxy- benzene group in this heterocyclic ring structure, thus two possible structures **1** or **2** remained as options.

Penicilliumthiamine B (**3**) was obtained as a white and amorphous powder. Its molecular formula of C_16_H_14_N_2_O_2_S (suggesting eleven degrees of unsaturation) was determined by HRESIMS (*m*/*z* 299.0853 [M + H]^+^, calcd. for C_16_H_15_N_2_O_2_S 299.0849). The characteristic NMR data of **3** closely resembled those of **1** and **2**, except for two sets of 4-hydroxybenzene groups and a thiadiazole group in **3** (Table 1, Figure 2). The locations of heteroatoms (S and N) could not be exactly assigned in these isomers, thus two possible structures **3** or **4** were also possible as the exact structure.

As limited quantities of penicilliumthiamine A and B were accessible through isolation from the organism, this piqued our interest in developing a total synthesis of the two pairs of thiazoles and thiadiazoles **1**–**4**, not only to facilitate the unambiguous confirmation of their initially uncertain structures, but also to support further biological evaluations and to enable structure-activity studies.

**Scheme 1:**
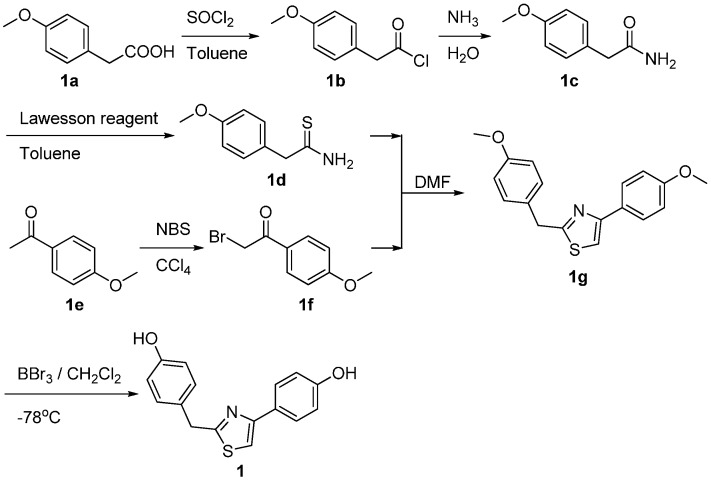
The synthetic route to **1**.

Compound **1** was prepared from the commercial availably 2-(4-methoxyphenyl)acetic acid (**1a**) and 1-(4-methoxyphenyl)ethanone (**1e**). Compound **1a** was halogenated, aminated and thiolated to generate the 2-(4-methoxyphenyl)ethanethioamide (**1d**) with Lawesson’s reagent (LR), which was cyclized with 2-bromo-1-(4-methoxyphenyl)ethanone (**1f**) to give 2-(4-methoxybenzyl)-4-(4-methoxyphenyl)thiazole (**1g**) according to the classical Hantzsch thiazole synthesis procedure. Finally, the methyl ether was successfully removed by boron tribromide (BBr_3_) as deprotection reagent, and 4-(2-(4-hydroxybenzyl)thiazol-4-yl)phenol (**1**) was successful synthesized in a total yield of 58% (Scheme 1).

All the important intermediates and products were confirmed by spectroscopic analysis with satisfactory spectral data. The important intermediate **1g** was also confirmed by the X-ray crystallographic analysis (CCDC number: 1434555). The ORTEP drawing of 1g with common atom numbering scheme was shown in Figure S21, Supplementary Materials. Although the exact structure of **1** was determined by spectroscopic analysis, it was regrettable that both its HPLC retention time and ^13^C-NMR signals were not consistent with those of penicilliumthiamines A.

**Scheme 2:**
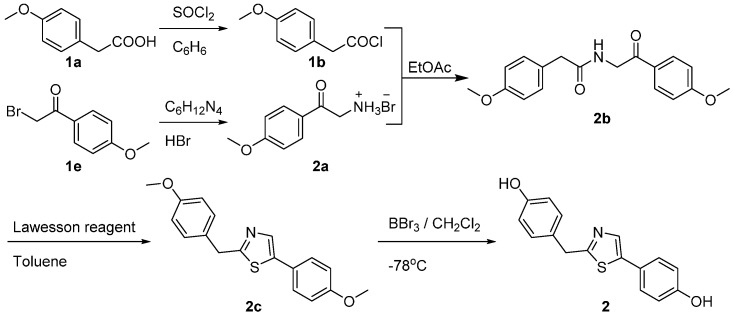
The synthetic route to **2**.

Compound **2** was also prepared from 4-methoxyphenylacetic acid (**1a**) and 2-bromo-1-phenylethanone (**1e**). Firstly, compound **1e** was aminated through a Delepine reaction to generate the 2-amino-1-(4-methoxyphenyl)ethanone (**2a**), which was acylated by 2-(4-methoxy-phenyl)acetyl chloride (**1b**) to give 2-(4-methoxyphenyl)-*N*-(2-(4-methoxyphenyl)-2-oxoethyl)-acetamide (**2b**). Then the amide **2b** smoothly underwent thiolation and subsequent cyclization by the action of Lawesson’s reagent in refluxing toluene to lead to thiazole **2c** by referring to recent literature [23,24]. Finally, complete removal of the methyl protection was affected with BBr_3_ at −78 °C to produce the target compound **2** in total 34% yield (Scheme 2). The structure of **2** was established by the spectroscopic analysis and X-ray crystallographic analysis (CCDC number: 1434559) conducted with colorless crystals grown from ethyl acetate (Figure S22, Supplementary Materials). It was exciting that the HPLC retention time and all the NMR signals of **2** completely matched with those of penicilliumthiamine A, therefore the structure of penicilliumthiamine A was finally confirmed.

**Scheme 3:**
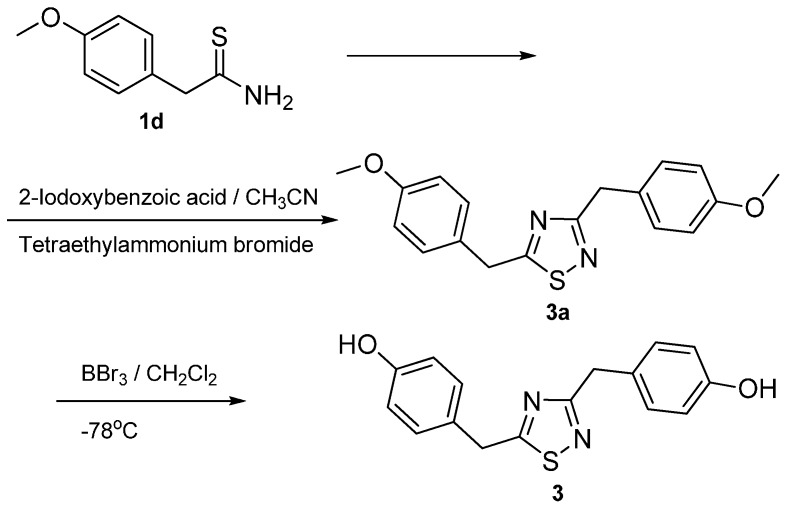
The synthetic route to **3**.

Compound **3** was synthesized by the oxidative dimerization of thioamides according to the Patil method (Scheme 3) [25]. The thioamide **1d** underwent oxidative dimerization by hypervalent iodine (V)-containing reagents, *o*-iodoxybenzoic acid (IBX) in the presence of tetraethylammonium bromide (TEAB) to generate the thiadiazole skeleton **3a**. The demethylation reaction was conducted by treating **3a** with BBr_3_ to give one target compound **3** in a total yield of 65% (Scheme 3). Compound **4** isomer was prepared by amination of **1a** with hydrazine, and the product bisacylhydrazine **4a** was cyclized to form the 1,3,4-thiadiazole core **4b** by Gierczyk’s method [26].

**Scheme 4:**
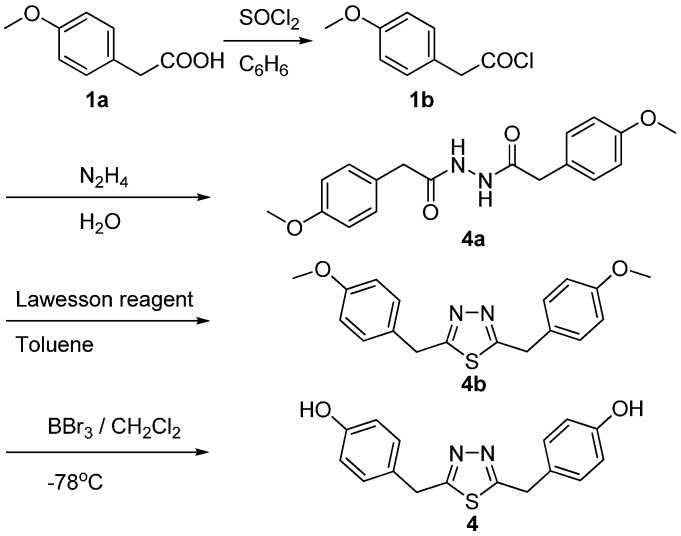
The synthetic route to **4**.

After deprotection of the methyl group, the compound **4** was obtained with four steps in total 49% yield (Scheme 4). After characterizing the structures, compound **3** was found to be identical to penicilliumthiamine B through comparison of the corresponding HPLC retention time and NMR signals.

**Figure 3:**
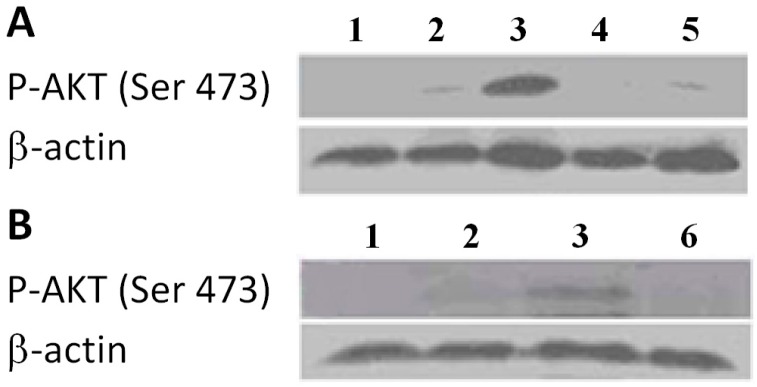
The phosphorylation of AKT/PKB (Ser 473) could be inhibited by compound **1**, **3a** and **3** (**A**) Representative western blot bands of P-AKT in MDA-MB-231 cells, (**B**) Representative western blot bands of P-AKT in HGC-27 cells; 1: Control, 2: LY294002 stimulated by 20 µmol/L 20 min before FBS 20 min, 3: FBS incubated at FBS 20 min, 4–6: Compound **3a**, **1** and **3** stimulated by 10 µmol/L 2 min before FBS 20 min, respectively).

**Figure 4:**
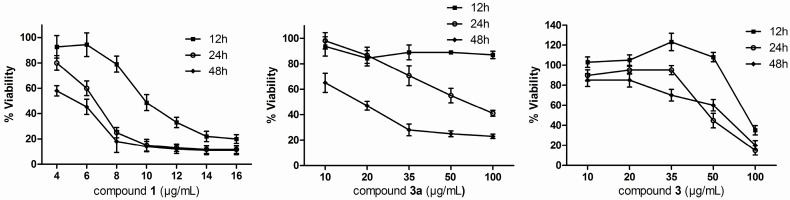
MTT assays showed the cytotoxicity on cancer cell lines.

All the compounds were firstly used to test whether they could inhibit the phosphorylation of AKT/PKB (Ser 473) under the stimulus of the fetal calf serum. The results showed that compounds **1** and **3a** could inhibit the phosphorylation of AKT/PKB (Ser 473) in the MDA-MB-231 cell while compound **3** inhibited the phosphorylation of AKT/PKB (Ser 473) in the HGC-27 cells (Figure 3). MTT experimental results showed that compound **1** exhibited the moderate growth inhibitory effect against MDA-MB-231 cell, which was in a dose- and time-dependent manner. The IC_50_ values of 12 h, 24 h, and 48 h for compound **1** in MDA-MB-231 cell were 37.16 µM, 22.36 µM, and 15.29 µM, respectively. Compounds **3a** and **3** showed certain inhibitory effect against MDA-MB-231 cell and HGC-27 cells, respectively. The IC_50_ values of compound **3a** were 199.30 µM and 51.80 µM against MDA-MB-231 cells for 24 h and 48 h. The IC_50_ values of 24 h and 48 h were 183.82 µM and 172.30 µM for compound **3** against HGC-27 cells, respectively (Figure 4). These results demonstrated that these cytotoxic compounds were cell-selective, and might target the phosphorylation of AKT/PKB (Ser 473), a key signaling component of one of the most frequently activated pathways in cancer and a major target of cancer drug development [27,28].

## 3. Experimental Section

### 3.1. General Procedures

UV spectra were run as methanol solutions on a Shimadzu UV-2550 spectrophotometer (Shimadzu, Kyoto, Japan). IR spectra were recorded on a Nicolet 380 FT-IR spectrophotometer (Thermo Fisher Scientific, Waltham, MA, USA). NMR spectra were recorded on Bruker AVANCE III 400 MHz Plus NMR spectrometer (Bruker, Bremen, Germany) using TMS as internal standard, Chemical shifts are reported as values and the coupling constants (*J*) are in Hz. HRESIMS spectra were got on a microTOF-QII mass spectrometer (Bruker, Bremen, Germany). Dionex UltiMate 3000 Rapid Separation LC Systems (Thermo Fisher Scientific, Waltham, MA, USA). A Cosmosil MS-II C18 preparative HPLC column (250 × 10 mm, 5 μm) was used. Column chromatography was carried out with silica gel (Qingdao Ocean Chemical Croup Co., Qingdao, China; 200–300 mesh), RP-C18 silica gel (YMC, Kyoto, Japan; 100–200 mesh), and Sephadex LH-20 (Amersham Biosciences, GE Healthcare Life Science, Santa Clara, CA, USA). The single-crystal X-ray diffraction analysis was performed on a Rigaku Mecury CCD diffractometer (Rigaku, Tokyo, Japan).

### 3.2. Fungal Material

The fungus *Penicillium oxalicum* was isolated from *Acrida cinerea* gut collected in July 2012 from the Chinese Big-Nine-Lake National Wetland Park in Hubei Province. The procedures of isolation and identification of the fungal strain used in this experiment were described in an earlier study [29]. The fungus was identified using a molecular biological protocol by DNA amplification and sequencing of the ITS region, as described in an earlier study [30]. The BLAST results indicated the sequence was the most similar (99%) to the sequence of *Penicillium oxalicum*. The strain was kept in the Hubei Key Laboratory of Natural Products Research and Development, China Three Gorges University.

### 3.3. Fermentation, Extraction and Isolation

The fermentation was carried out dynamically in a SD medium (consisting of 40 g glucose, 10 g peptone in 1 L of distilled water) in 500 mL Erlenmeyer flasks for 20 days at room temperature. The fermented liquids substrate (200 flasks) was extracted repeatedly with ethyl acetate, and the organic layers were combined and evaporated to dryness under vacuum to afford an extract (13.0 g), which was fractionated by silica gel chromatography using chloroform–methanol (100:0–50:50, *v*/*v*) gradient elution to produce five portions (Fr. I–Fr. V). Fractions III were combined and subjected to silica gel column chromatography, Sephadex LH-20 gel, and preparative reverse-phase C_18_ HPLC (250 × 10 mm i.d., Cosmosil MS-II) using an acetonitrile–water system (27:73, *v*/*v*) to yield compound **1** or **2** (4.1 mg) and **3** or **4** (5.5 mg).

### 3.4. Synthesis

*2-(4-Methoxyphenyl)ethanethioamide* (**1d**). In a round bottomed flask equipped with a magnetic stirring bar and argon gas inlet, 2-(4-methoxyphenyl)acetic acid (**1a**, 5.0 mmol) was dissolved in thionyl chloride (10.0 mL). The mixture was allowed to heat to reflux for 3 h, which was concentrated to remove the additional thionyl chloride under reduced pressure to give the 2-(4-methoxyphenyl)acetyl chloride (**1b**). Then ammonia solution (5.0 mmol ammonia gas in 5.0 mL water) was added into the acyl chloride in ethyl acetate (5.0 mL), and the 2-(4-methoxyphenyl)acetamide (**1c**) was crystallize form the mixture in 95% yield as white solids; Its spectral data were identical with those reported with the melting point of 169–170 °C (lit. [31] 163–165 °C). The 2-(4-methoxyphenyl)ethanethioamide (**1d**, 0.45 g) was synthesized and isolated by using Lawesson's reagent (5.0 mmol) in toluene (20.0 mL) under 50 °C for 2 h, and the residue was purified by flash column chromatography on silica gel (EtOAc/hexane, 1:1) to afford yellow solids with the yield of 85%. ^1^H-NMR (400 MHz, DMSO-*d*_6_, δ ppm): 7.66 (s, 1H), 7.26–7.17 (m, 2H), 6.92–6.71 (m, 2H), 6.70 (s, 1H), 4.05 (s, 2H), 3.81 (s, 3H).

*2-Bromo-1-(4-methoxyphenyl)ethanone* (**1f**). 1-(4-Methoxyphenyl)ethanone (**1e**, 5.0 mmol) and *N*-bromosuccinimide (NBS, 5.0 mmol) were stirred in carbon tetrachloride (CCl_4_, 20.0 mL) at 50 °C for 2 h. After **1e** was completely consumed, the succinimide was removed by filtration. The organic phase was washed with water (10 mL), dried over Na_2_SO_4_ and solvent evaporated under reduced pressure to give the 2-bromo-1-(4-methoxyphenyl)ethanone (**1f**). White solid, yield 90%, mp 70–72 °C (lit. [32] 69–71 °C). ^1^H-NMR (CDCl_3_, δ ppm): 7.97 (d, *J* = 8.8 Hz, 2H), 6.96 (d, *J* = 8.8 Hz, 2H), 4.40 (s, 2H), 3.89 (s, 3H). ^13^C-NMR (CDCl_3_, δ ppm): 189.94, 164.11, 131.34, 126.87, 114.04, 55.55, 30.69.

*2-(4-Methoxybenzyl)-4-(4-methoxyphenyl)thiazole* (**1g**). Compound **1f** (2.0 mmol) was heated with **1d** (2.0 mmol) in *N*,*N*′-dimethylformide (DMF, 20.0 mL) at 100 °C for 5 h under the protection of nitrogen atmosphere. The progress of the reaction was monitored by thin-layer chromatography (TLC). After disappearance of starting materials, the solution was cooled to room temperature, and 10% NaCl solution (80.0 mL) was added, and then the product was extracted with dichloromethane (40.0 mL × 3). The organic phase was separated, washed with saturated NaCl solution (50 mL × 2) and dried over anhydrous sodium sulphate. Removal of the solvent on a rotary evaporator under high vacuum gave a viscous brown oil, which was purified by flash column chromatography on silica gel (eluent: *n*-hexane/EtOAc = 5:1, *v*/*v*) to yield compound **1g** as a white solid in 80% yield, mp 118–120 °C. IR (KBr, cm^−1^): = 3107, 2956, 2937, 2838, 1687, 1608, 1582, 1530, 1511, 1492, 1466, 1453, 1440, 1419, 1323, 1299, 1275, 1252, 1207, 1171, 1109, 1055, 1028, 989, 850, 835. ^1^H-NMR (DMSO-*d*_6_, δ ppm): 7.82 (dd, *J* = 6.8, 2Hz, 2H), 7.29–7.26 (t, *J* =8.8, 4Hz, 2H), 7.19 (s, 1H), 6.94 (dd, *J* = 6.8, 2.0 Hz, 2H), 6.88 (dd, *J* = 6.4, 2.0 Hz, 2H), 4.31 (s, 2H), 3.85 (s, 3H), 3.81 (s, 3H). ESIMS *m*/*z*: 312 [M + H]^+^.

*2-(4-Methoxyphenyl)-2-oxoethanaminium bromide* (**2a**). 2-Bromo-1-(4-methoxyphenyl)ethanone (**1e**, 10.0 mmol) was added to a solution of hexamethylenetetramine (1.40 g, 10.0 mmol) in chloroform (40.0 mL), and the resulting mixture was heated at 50 °C for 3 h. The mixture was cooled to room temperature and the white precipitate was collected by filtration, washed with CHCl_3_, and dried *in vacuo* to afford **2a** as white solids, yield 90%, mp 191–193 °C (lit. [33] 195–197 °C). ^1^H-NMR (DMSO-*d*_6_, δ ppm): 8.46 (br s, 3H), 7.99 (dd, *J* = 9.2, 2.0 Hz, 2H), 7.09 (dd, *J* = 9.2, 2.0 Hz, 2H), 4.49 (s, 2H), 3.86 (s, 3H). ^13^C-NMR (DMSO-*d*_6_, δ ppm): 191.1, 146.1, 130.6, 126.6, 114.2, 55.7, 44.3.

*2-(4-Methoxyphenyl)-N-(2-(4-methoxyphenyl)-2-oxoethyl)acetamide* (**2b**). 2-(4-Methoxyphenyl)acetyl chloride (**1b**) was dissolved in anhydrous ethyl acetate (20.0 mL) under 0 °C, then **2a** (5.0 mmol) was added and the amide **2b** was quickly precipitated in 5 min. The yellow solids were collected by filtration, washed with ethyl acetate (2 × 8.0 mL) and dried under vacuum to afford analytically pure product in 50% yield, as a white solid, mp 83–86 °C. ^1^H-NMR (DMSO-*d*_6_, δ ppm): 7.91 (d, *J* = 8.8 Hz, 2H), 7.24 (d, *J* = 8.4 Hz, 2H), 6.95–6.90 (m, 4H), 6.58 (s, 1H), 4.67 (d, *J* = 4.0 Hz, 2H), 3.87 (s, 3H), 3.81 (s, 2H), 3.61 (s, 2H). ^13^C-NMR (DMSO-*d*_6_, δ ppm): 192.37, 171.54, 164.23, 158.86, 130.49, 130.17, 127.27, 126.51, 114.40, 114.07, 55.52, 55.23, 46.03, 42.71.

*2-(4-Methoxybenzyl)-5-(4-methoxyphenyl)thiazole* (**2c**). To a solution of the above amide **2b** (2.0 mmol) in toluene (20.0 mL) was added Lawesson’s reagent (10.0 mmol), and the mixture was heated under reflux for 2 h. The solvent was evaporated under reduced pressure, and the residue was purified by silica gel column chromatography (petroleum ether: ethyl acetate = 1:1, *v*/*v*) to give the thiazole **2c** as a white solid in 85% yield, mp 111–112 °C. IR (KBr, cm^−1^): 2963, 2836, 1609, 1536, 1514, 1501, 1466, 1424, 1307, 1302, 1283, 1249, 1212 1183, 1174, 1108, 1071, 1030, 843, 825. ^1^H-NMR (CDCl_3_, δ ppm): 7.72 (s, 1H), 7.41–7.39 (m, 2H), 7.28-7.26 (m, 2H), 6.90–6.87 (m, 4H), 4.25 (s, 2H), 3.82 (s, 3H), 3.80 (s, 3H). ^13^C-NMR (CDCl_3_, δ ppm): 169.58, 159.51, 158.70, 139.18, 136.76, 130.07, 130.01, 127.83, 124.15, 114.35, 114.17, 55.32, 55.24, 51.59, 39.03, 30.90. ESIMS *m*/*z* = 312 [M + H]^+^.

*3,5-Bis(4-methoxybenzyl)-1,2,4-thiadiazole* (**3a**). To a stirred suspension of IBX (3.5 mmol) and TEAB (3.5 mmol) in acetonitrile (20.0 mL) was added 2-(4-methoxyphenyl)ethanethioamide (**1d**) in 20 min at 10 °C. Consumption of starting material was observed by TLC. After completion of reaction, acetonitrile was removed under reduced pressure and the resultant residue was washed with ethyl acetate (25.0 mL) followed by 10% sodium bisulfite solution (30.0 mL), saturated sodium carbonate (30.0 mL), and brine (30.0 mL). The organic layer was dried over anhydrous sodium sulfate and concentrated under reduced pressure to give crude product. Pure product was isolated after column chromatography on silica gel mesh (eluent: petroleum ether/ethyl acetate = 2/1, *v*/*v*) to yield the compound **3a** as white solids in 90% yield, mp 69–71 °C. IR (KBr, cm^−1^): 2933, 2840, 1609, 1582, 1512, 1491, 1456, 1444, 1432, 1301, 1283, 1247, 1221, 1178, 1146, 1116, 1087, 1026, 837, 814. ^1^H-NMR (CDCl_3_, δ ppm): 7.27–7.21 (m, 2H), 6.89 (m, 2H), 4.27 (s, 2H), 4.23 (s, 2H), 3.81 (s, 3H), 3.78 (s, 3H). ^13^C-NMR (CDCl_3_, δ ppm): 193.25, 175.99, 159.13, 158.40, 130.20, 130.06, 129.21, 128.19, 114.41, 113.99, 55.26, 55.21, 38.44, 37.01. ESIMS *m*/*z* = 327 [M + H]^+^.

*2-(4-Methoxyphenyl)-N*′*-(2-(4-methoxyphenyl)acetyl)acetohydrazide* (**4a**). 2-(4-Methoxyphenyl)acetyl chloride (**1b**, 5.0 mmol) was dissolved in anhydrous ethyl acetate (20.0 mL) under 0 °C, then 80% hydrazine was added. Then the bisacetohydrazide **4a** was immediately precipitated within 5 min. The white solids were collected by filtration, washed with ethyl acetate (2 × 10.0 mL) and dried under vacuum to afford analytically pure product in 90% yield, mp 108–109 °C. ^1^H-NMR (DMSO-*d*_6_, δ ppm): 7.16 (br s, 2H), 7.19 (d, *J* = 8.4 Hz, 4H), 6.85 (d, *J* = 8.4 Hz, 4H), 3.71 (s, 6H), 3.37 (s, 4H). ^13^C-NMR (DMSO-*d*_6_, δ ppm): 169.22, 157.96, 130.00, 127.63, 113.62, 55.02, 39.21.

*2,5-Bis(4-methoxybenzyl)-1,3,4-thiadiazole* (**4b**). To a solution of the above bisacetohydrazide **4a** (2.0 mmol) in toluene (20.0 mL) was added Lawesson’s reagent (10.0 mmol), and the mixture was heated at reflux for 4 h. The solvent was evaporated under reduced pressure, and the residue was purified by silica gel column chromatography (petroleum ether:ethyl acetate = 1:1, *v*/*v*) to give the thiadiazole **4b** as a white solid in 60% yield, mp 108–110 °C. IR (KBr, cm^−1^): 2960, 2916, 2837, 2360, 2341, 1611, 1584, 1513, 1456, 1442, 1426, 1301, 1251, 1214, 1175, 1140, 1118, 1032, 837, 825. ^1^H-NMR (CDCl_3_, δ ppm): 7.16 (d, *J* = 8.4 Hz, 4H), 6.83 (d, *J* = 8.4 Hz, 4H), 4.27 (s, 4H), 3.77 (s, 6H). ^13^C-NMR (CDCl_3_, δ ppm): 171.39, 158.86, 129.86, 129.20, 114.29, 55.23, 35.69. ESIMS *m*/*z* = 327 [M + H]^+^.

### 3.5. General Procedure for the Demethylation Reaction

A cooled solution of the methyl ether (**1f**, **2c**, **3a** or **4b**, 0.5 mmol) in dichloromethane (CH_2_Cl_2_, 5.0 mL) was treated with BBr_3_ (1.0 mmol BBr_3_ in 2.0 mL CH_2_Cl_2_), and then the mixture was allowed to stand at –78 °C for 3 h until the methyl ether was consumed completely (monitored by TLC). Finally the solution was diluted with 1% NaHCO_3_ (30.0 mL), and extracted with CH_2_Cl_2_ (3 × 20.0 mL). The organic layers were combined, dried over anhydrous Na_2_SO_4_, filtered and concentrated *in vacuo* to give a yellow residue. The crude product was purified by flash chromatography on silica gel to yield the target compound **1**–**4** (eluent: petroleum ether/ethyl acetate = 2/1, *v*/*v*).

*4-(2-(4-Hydroxybenzyl)thiazol-4-yl)phenol* (**1**). White and amorphous powder, yield 86%, mp 200–201 °C. IR (KBr, cm^−1^): 3389, 3103, 3009, 2951, 2792, 1610, 1594, 1515, 1489, 1438, 1375, 1273, 1244, 1213, 1182, 1170, 1133, 839. ^1^H-NMR (DMSO-*d*_6_, δ ppm): 9.60 (br s, 1H), 9.37 (br s, 1H), 7.75-7.73 (m, 2H), 7.65 (s, 1H), 7.15 (d, *J* = 8.4 Hz, 2H), 6.80-6.79 (m, 2H), 6.74–6.71 (m, 2H), 4.21 (s, 2H). ^13^C-NMR (DMSO-*d*_6_, δ ppm): 170.7, 157.3, 156.3, 154.3, 130.0, 128.3, 127.3, 125.6, 115.4, 115.3, 111.0, 49.1, 39.9, 39.7, 39.5, 39.3, 39.1, 38.1, 38.0. HRESIMS, calcd. for C_16_H_14_NO_2_S [M + H]^+^ 284.0745, found 284.0749.

*Penicilliumthiamine A* (**2**). White and amorphous powder, yield 90%, mp 189–190 °C. IR (KBr, cm^−1^): 3238, 3018, 2961, 2925, 2668, 1609, 1596, 1586, 1515, 1504, 1447, 1422, 1375, 1278, 1244, 1176, 1103, 1082, 1030, 852, 832. ^1^H- and ^13^C-NMR data, see Table 1. HRESIMS, calcd. for C_16_H_14_NO_2_S [M + H]^+^ 284.0745, found 284.0746.

*Penicilliumthiamine B* (**3**). White and amorphous powder, yield 90%, mp 180–181 °C. IR (KBr, cm^−1^): 3520, 3158, 1614, 1593, 1516, 1496, 1449, 1434, 1359, 1336, 1311, 1265, 1232, 1209, 1173, 1102, 842, 833. ^1^H- and ^13^C-NMR data, see Table 1. HRESIMS, calcd. for C_16_H_15_N_2_O_2_S [M + H]^+^ 2899.0854, found 299.0853.

*4,4*′*-((1,3,4-Thiadiazole-2,5-diyl)bis(methylene))diphenol* (**4**). White and amorphous powder, yield 90%, mp 209–210 °C. IR (KBr, cm^−1^): 3520, 3158, 1614, 1593, 1516, 1496, 1449, 1434, 1311, 1265, 1232, 1209, 1173, 1102, 816 cm-1. ^1^H-NMR (DMSO-*d*_6_, δ ppm): 9.37 (br s, 2H), 7.07 (d, *J* = 8.4 Hz, 4H), 6.71–6.67 (m, 4H), 4.20 (s, 4H). ^13^C-NMR (DMSO-*d*_6_, δ ppm): 171.07, 156.42, 129.83, 127.84, 115.51, 34.45. HRESIMS, calcd. for C_16_H_15_N_2_O_2_S [M + H]^+^ 2899.0854, found 299.0859.

### 3.6. Biological Evaluation

Each compound was dissolved in distilled water. The filtered stock compound solution was separated into individual aliquots which were kept at −20 °C until further use. Human breast cancer MDA-MB-231 cells and human gastric cancer HGC-27 cells were from the Institute of Molecular Biology, China Three Gorges University. Cancer cells were maintained in RPMI 1640 culture medium supplemented with 10% fetal bovine serum and antibiotics in a 5% carbon dioxide incubator at 37 °C. All the cells were firstly starved for 12 h and stimulated by FBS 20 min before adding the drugs for 2 min. A western blot assay was used to detect the phosphorylation of PKB/AKT (Ser473) kinase. Then analyze the cytotoxicity of positive compounds on cancer cell lines, cells were treated with different concentrations at different time points respectively (MTT assay).

## 4. Conclusions

In summary, in this work four total synthetic routes were designed to prepare thiazoles and thiadiazoles using commercial 1-(4-methoxyphenyl)-ethanone and 4-methoxyphenylacetic acid as the starting materials, and two of them were confirmed as penicilliumthiamines A and B (compounds **2** and **3**) from the extract of the *Penicillium oxalicum*. Compounds **1**, **3a** and **3** showed different cytotoxicity activities with cell selectivity, which might all target AKT/PKB. This is the first report on the anticancer activities of these new molecules, which could be the starting point for further development of drug candidates with potential in the treatment of cancer.

## Figures and Tables

**Table 1:** ^1^H-NMR (400 MHz) and ^13^C-NMR (100 MHz) data of **2** and **3** (DMSO-*d*_6_).

2			3		
No	δ_H_ (*J* in Hz)	δ_C_	No	δ_H_ (*J* in Hz)	δ_C_
1	-	-	1	-	-
2	-	168.66	2	-	193.51
3	-	-	3	-	-
4	7.84, s	136.43	4	-	175.75
5	-	138.76	5	-	-
7’	4.15, s	38.03	7’	4.28, s	35.79
-			7”	4.09, s	37.63
1’	-	128.36	1’	-	126.89
2’	7.13, d (8.6)	129.99	2’	7.16, d (8.4)	130.21
3’	6.72, d (8.6)	115.38	3’	6.73, d (8.4)	115.54
4’	-	156.29	4’	-	156.64
5’	6.72, d (8.6)	115.38	5’	6.73, d (8.4)	115.54
6’	7.13, d (8.6)	129.99	6’	7.16, d (8.4)	130.21
1”	-	121.92	1”	-	127.42
2”	7.37, d (8.7)	127.63	2”	7.07, d (8.5)	129.86
3”	6.77, d (8.7)	115.89	3”	6.67, d (8.5)	115.14
4”	-	157.54	4”	-	155.95
5”	6.77, d (8.7)	115.89	5”	6.67, d (8.5)	115.14
6”	7.37, d (8.7)	127.63	6”	7.07, d (8.5)	129.86

## Supplementary Materials

Supplementary materials can be accessed at: http://www.mdpi.com/1420-3049/21/3/232/s1.
