# Supplementary material for: Cytotoxic 1,3-Thiazole and 1,2,4-Thiadiazole Alkaloids from Penicillium oxalicum: Structural Elucidation and Total Synthesis
Source: Molecules. 2016 Feb 26;21(3):232. doi: 10.3390/molecules21030232 (PMC6273271; doi:10.3390/molecules21030232)
Supplement: Supplementary file 1 [file molecules-21-00232-s001.pdf]

Supplementary Materials: Cytotoxic 1,3-Thiazole and 1,2,4-Thiadiazole Alkaloids from *Penicillium oxalicum*: Structural Elucidation and Total Synthesis

Zheng Yang, Nianyu Huang, Bang Xu, Wenfeng Huang, Tianpeng Xie, Fan Cheng and Kun Zou

Table of Contents

1. ^{1}H-NMR and ^{13}C-NMR Spectra for the compounds **1g, 1, 2c, 2, 3a, 3, 4b, 4** S2

2. HPLC and ^{13}C-NMR comparing analysis S11

3. The crystal structure of **1g** with numbered atoms S14

4. The crystal structure of **2** with numbered atoms S14

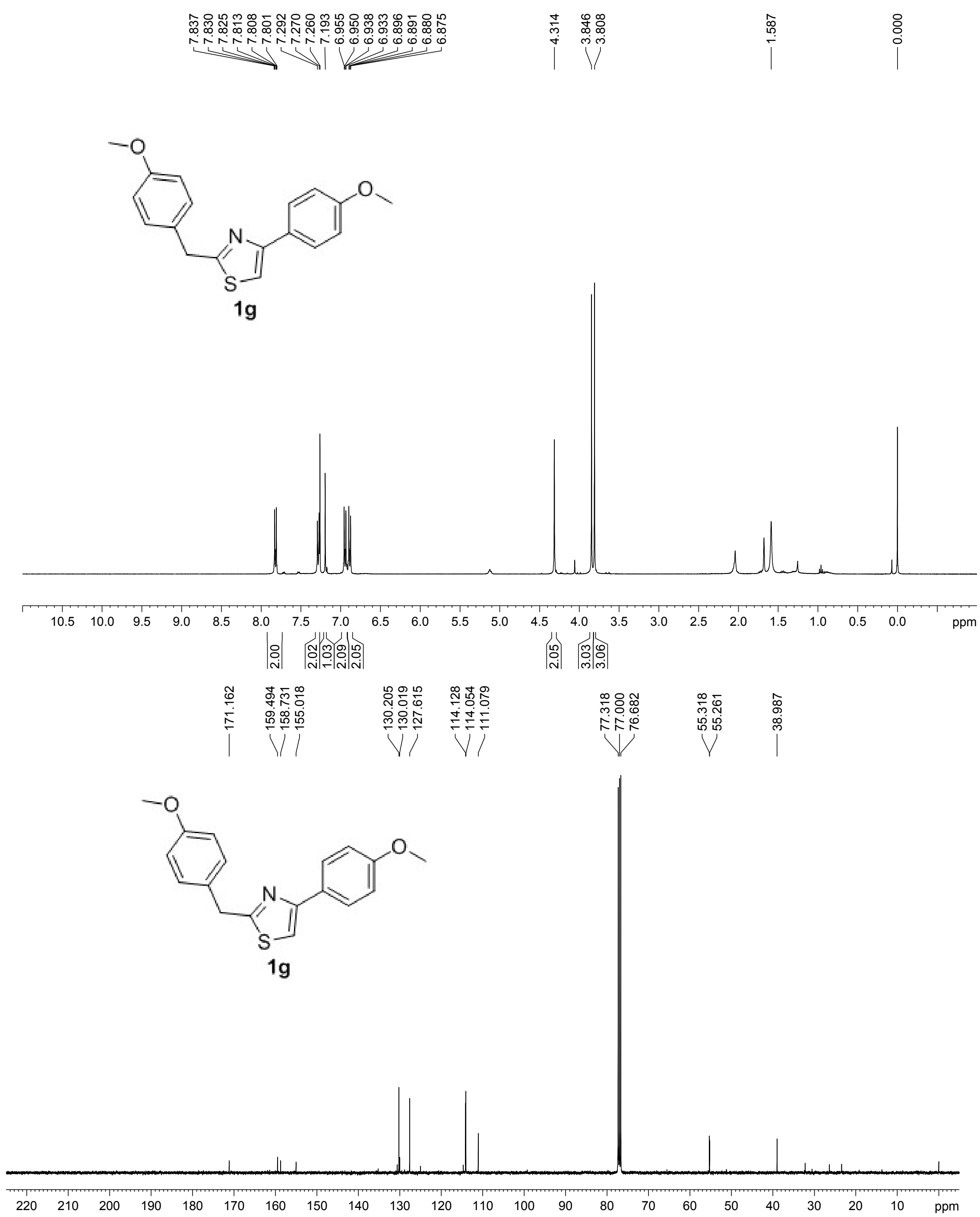

Figure S1. ^{1}H-NMR and ^{13}C-NMR of compound **1g**.

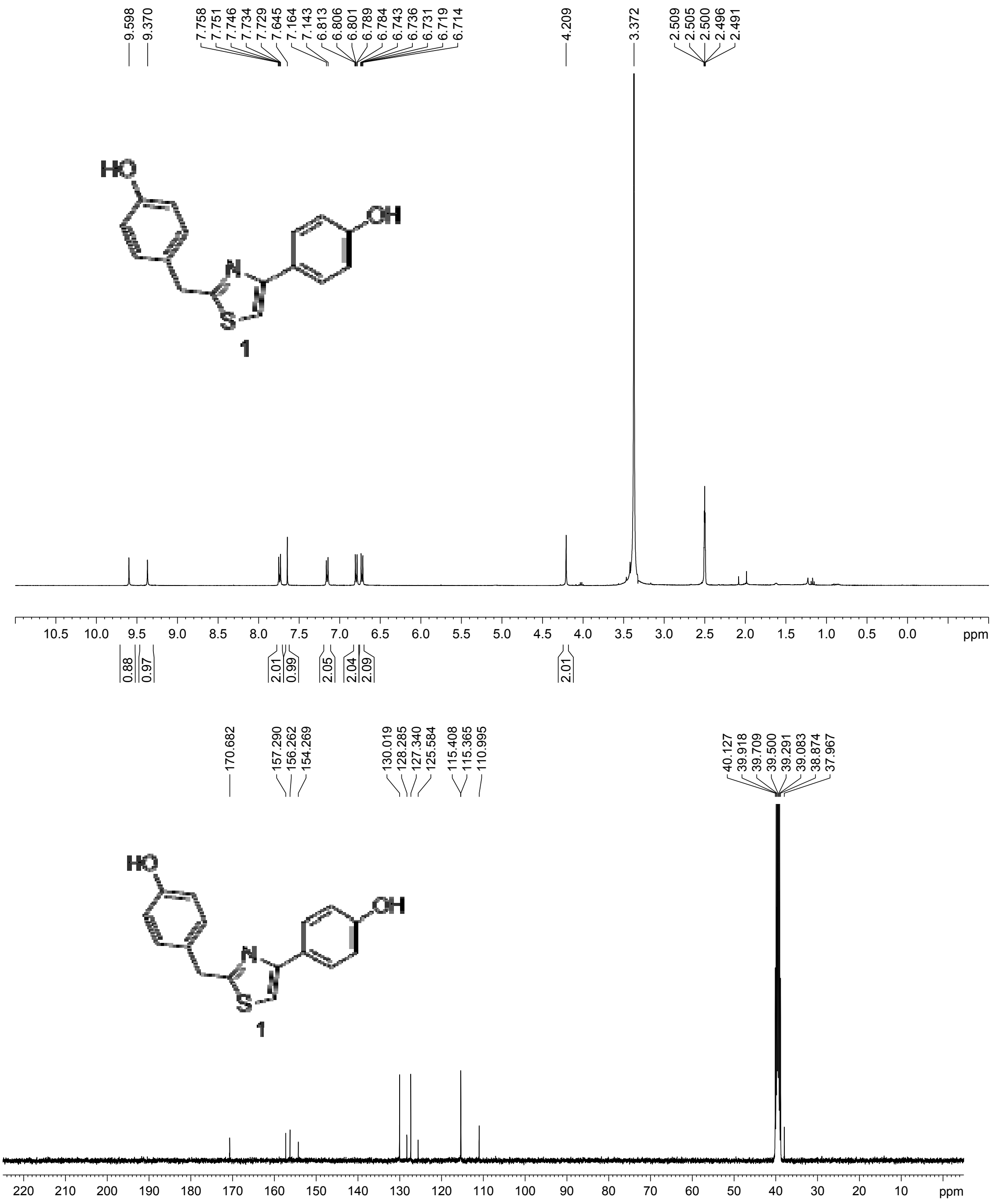

Figure S2. ^{1}H-NMR and ^{13}C-NMR of compound **1**.

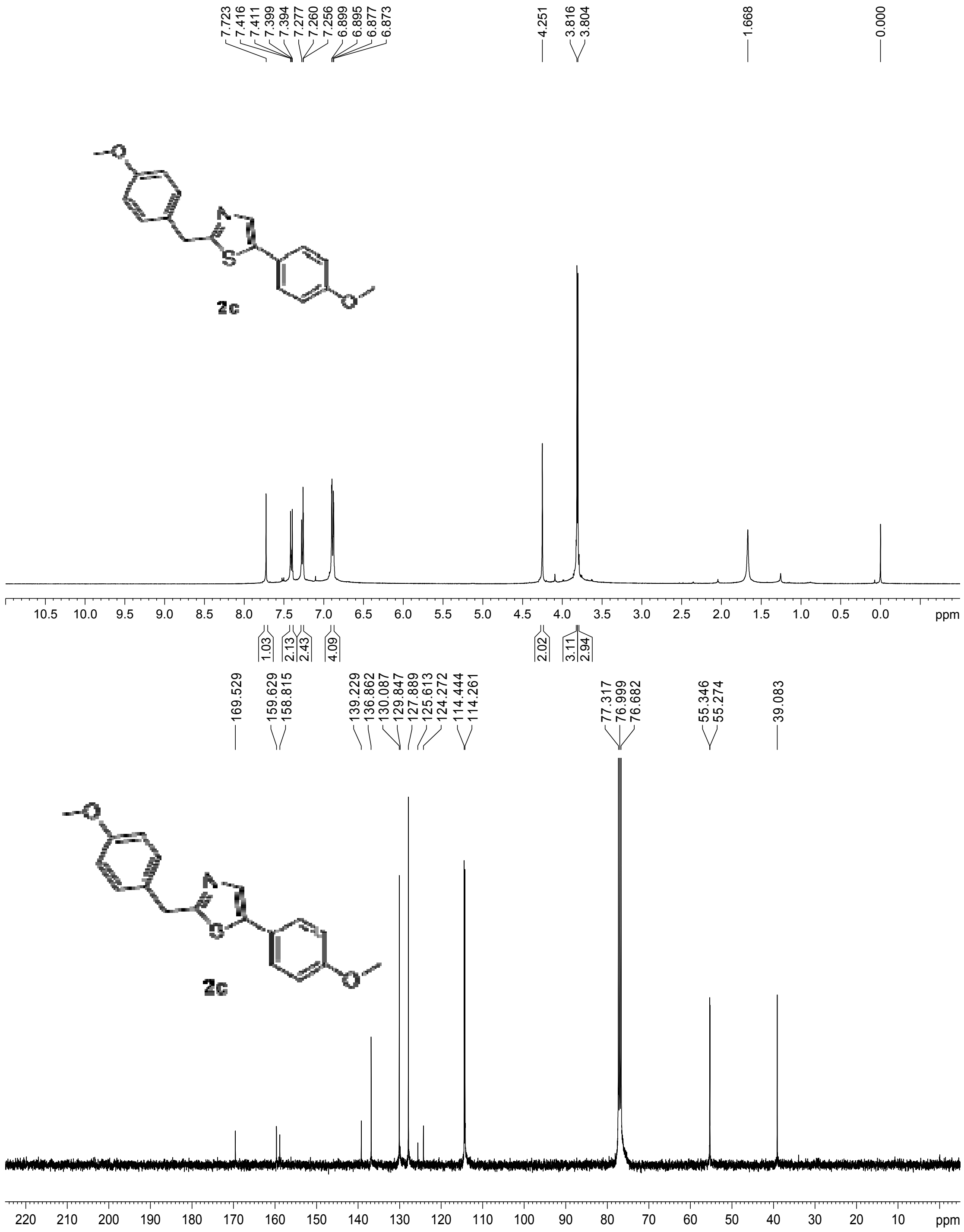

Figure S3. ^{1}H-NMR and ^{13}C-NMR of compound **2c**.

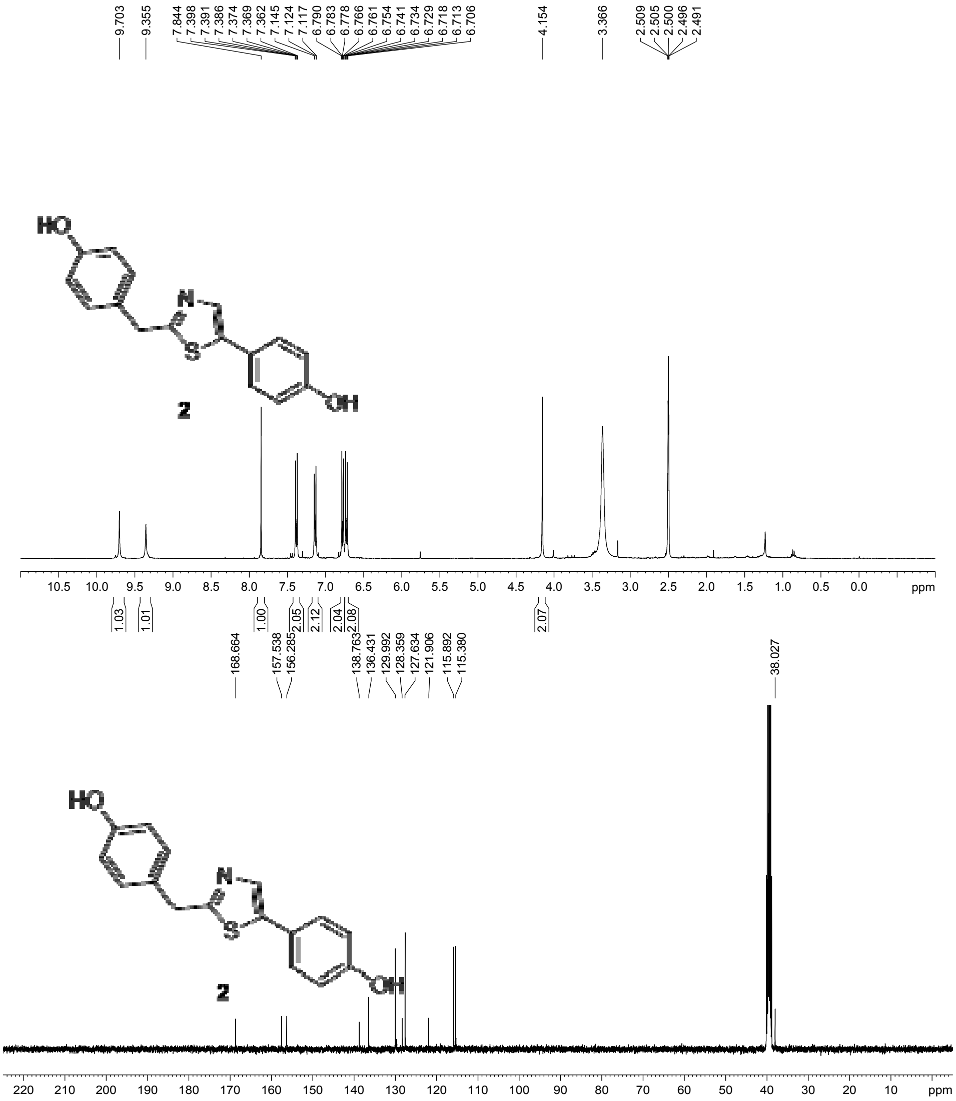

Figure S4. ^{1}H-NMR and ^{13}C-NMR of compound **2**.

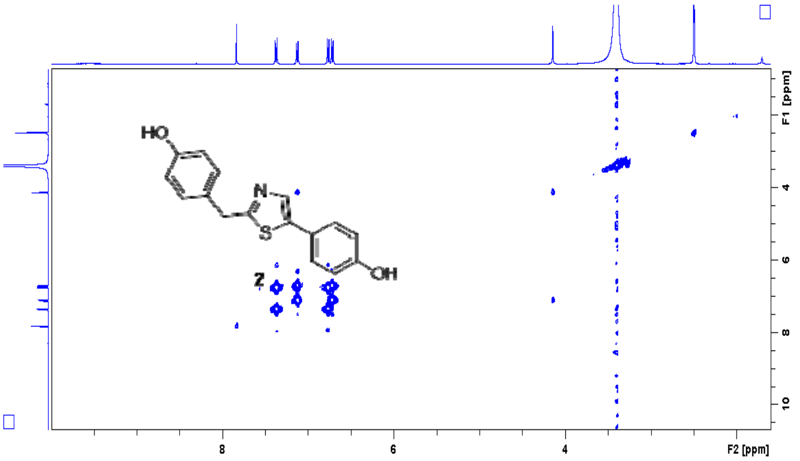

Figure S5. ^{1}H-^{1}H COSY of compound **2**.

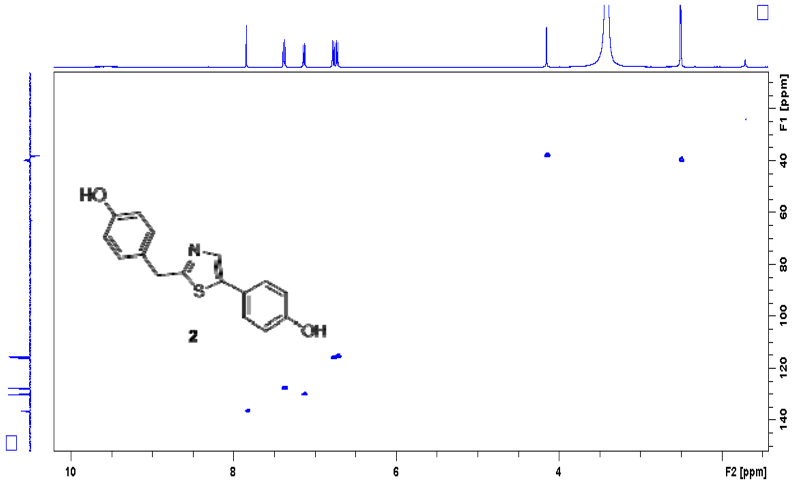

Figure S6. HSQC of compound **2**.

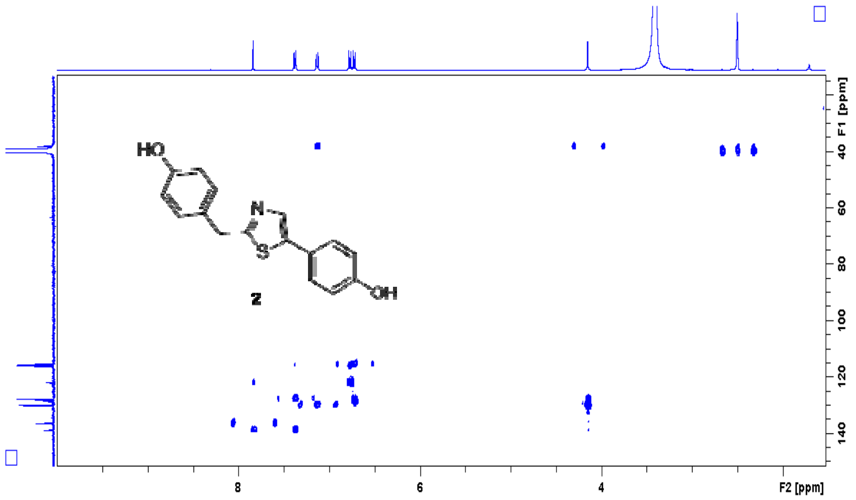

Figure S7. HMBC of compound **2**.

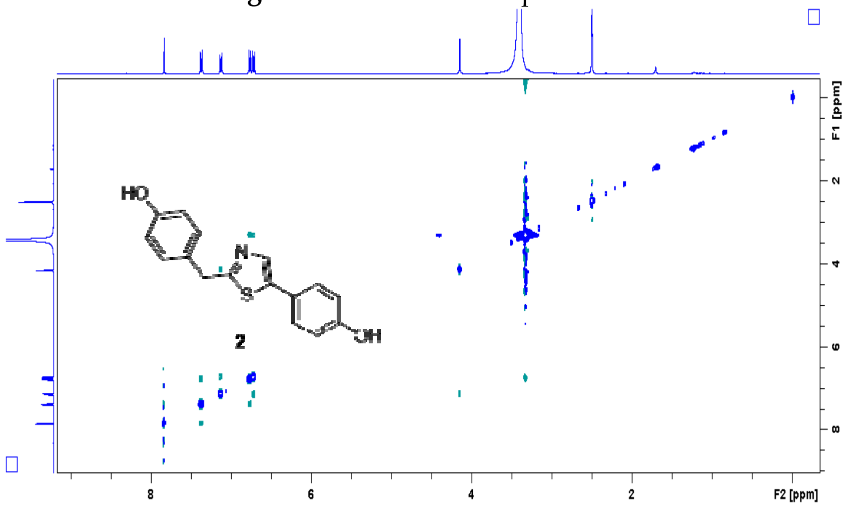

Figure S8. NOESY of compound **2**.

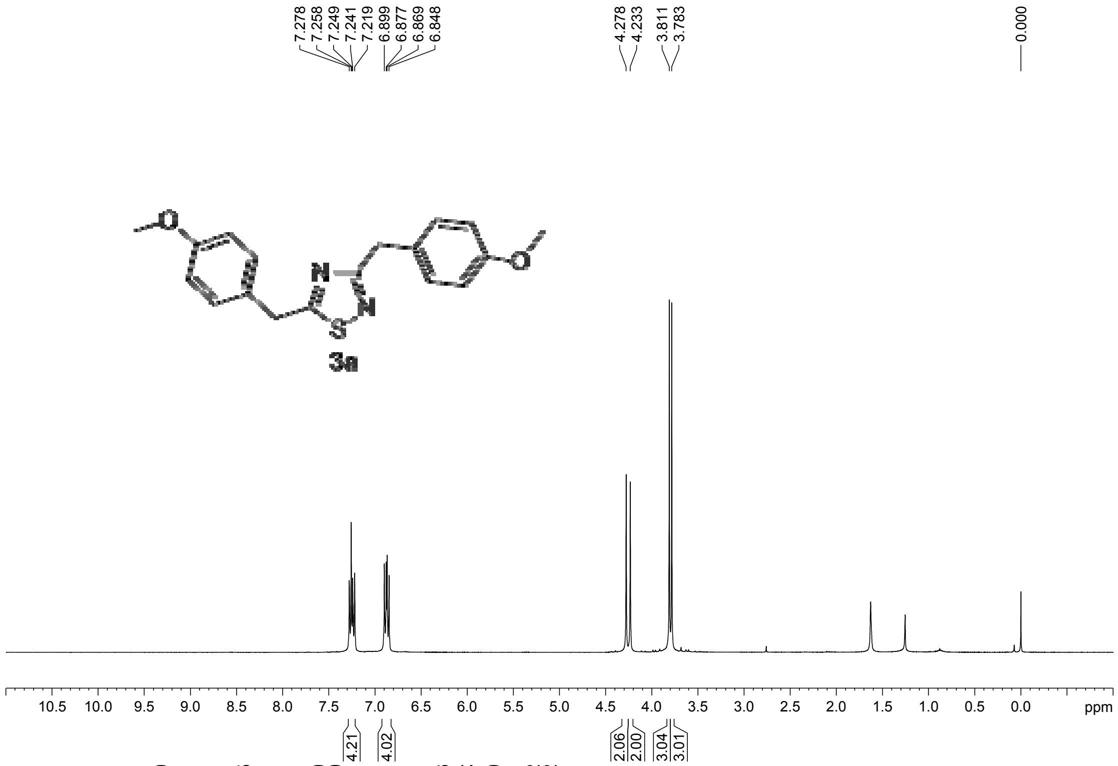

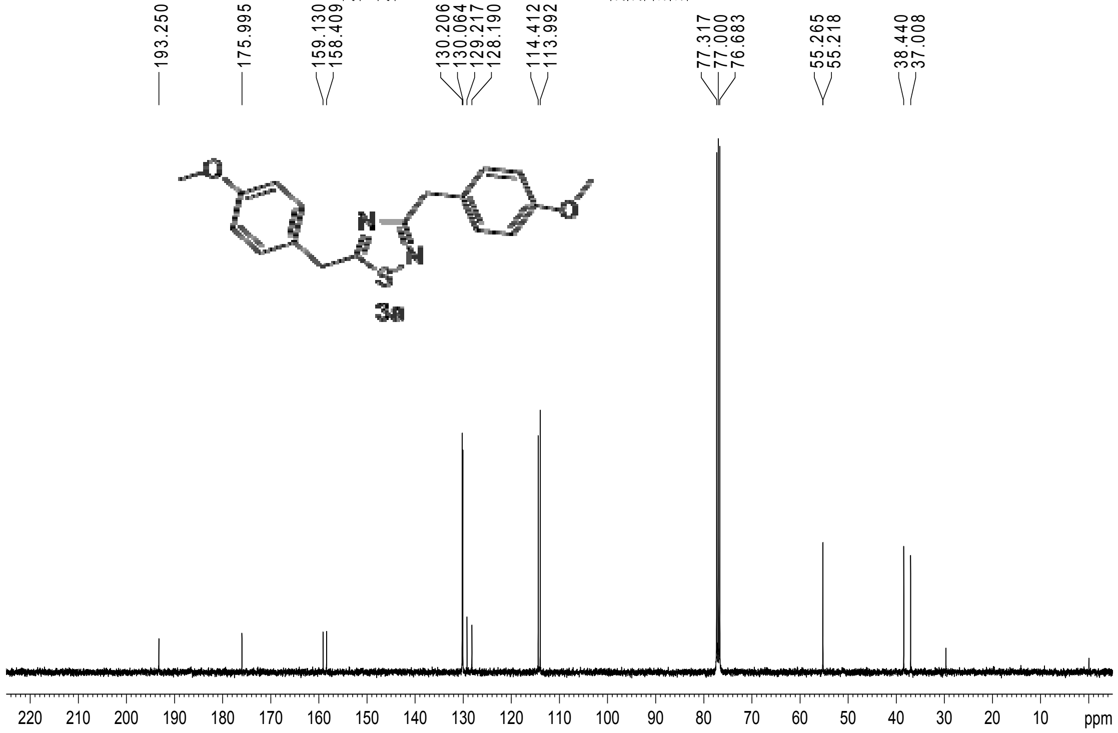

Figure S9. ^{1}H-NMR and ^{13}C-NMR of compound **3a**.

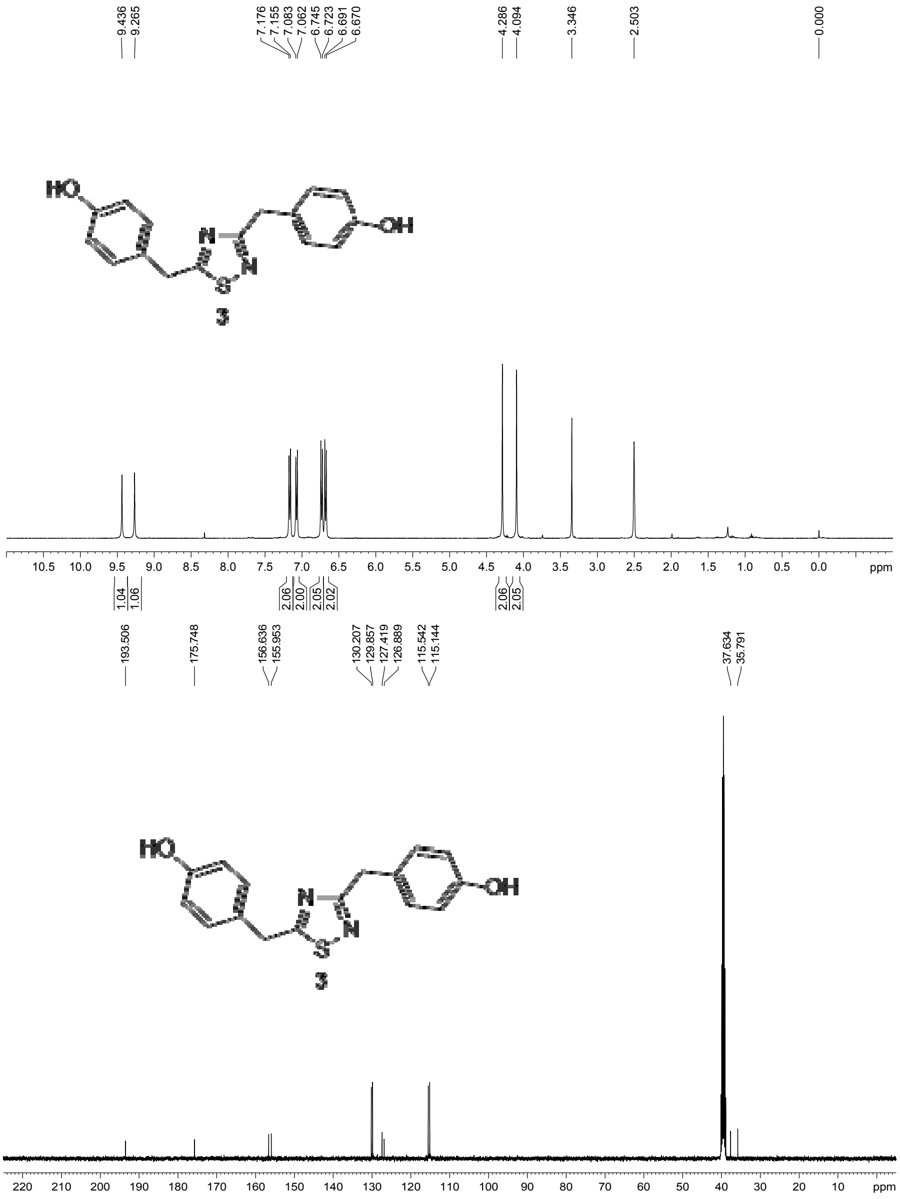

Figure S10. ^{1}H-NMR and ^{13}C-NMR of compound **3**.

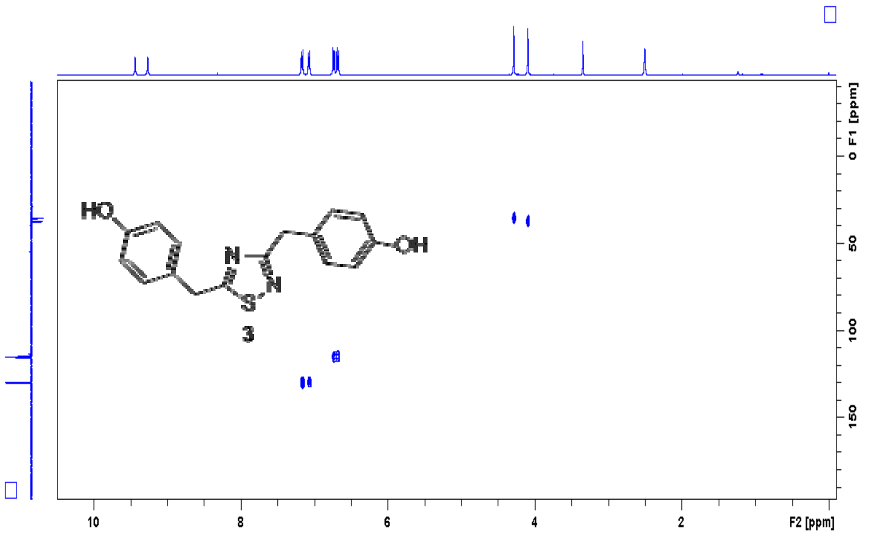

Figure S11. HSQC of compound **3**.

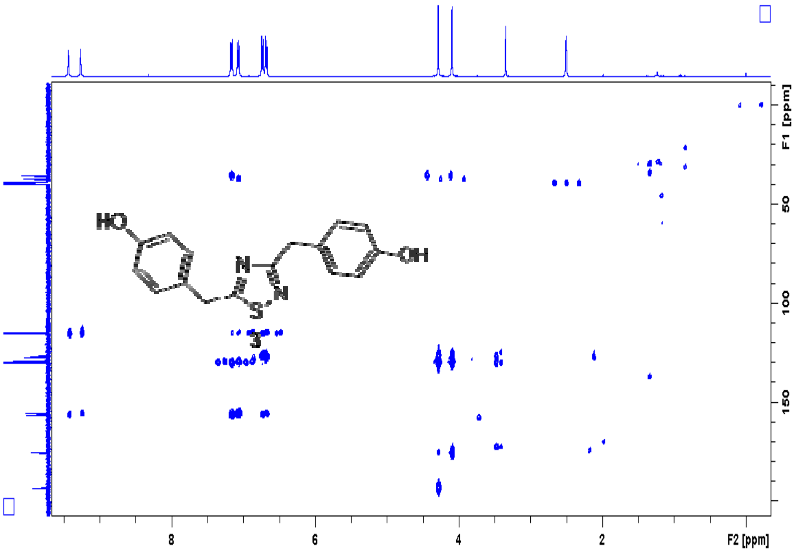

Figure S12. HMBC of compound **3**.

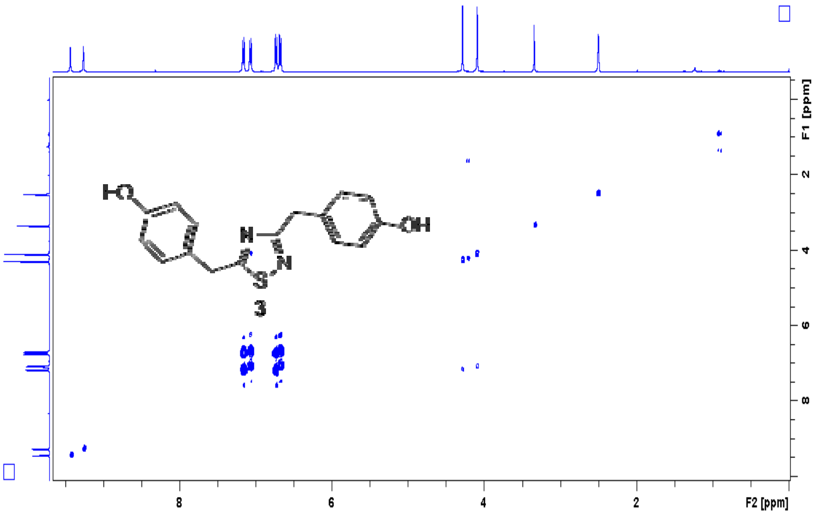

Figure S13. ^{1}H-^{1}H COSY of compound 3.

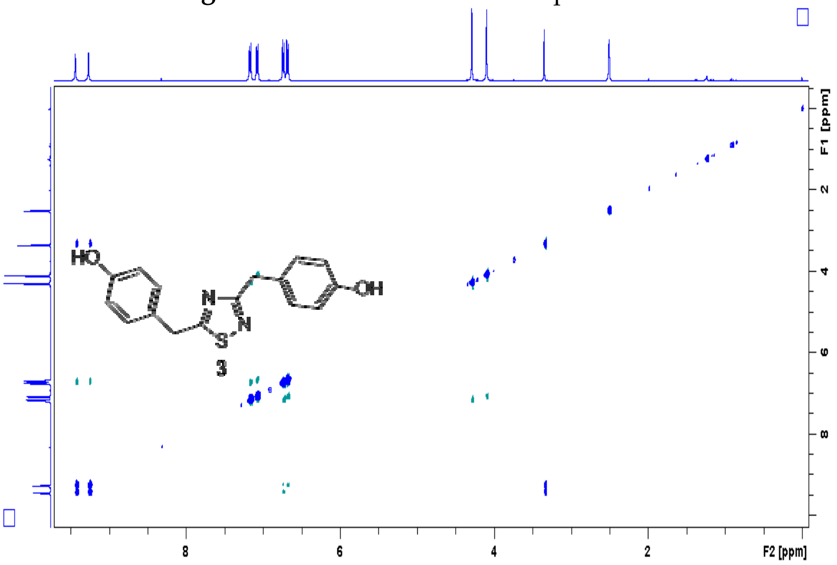

Figure S14. NOESY of compound **3**.

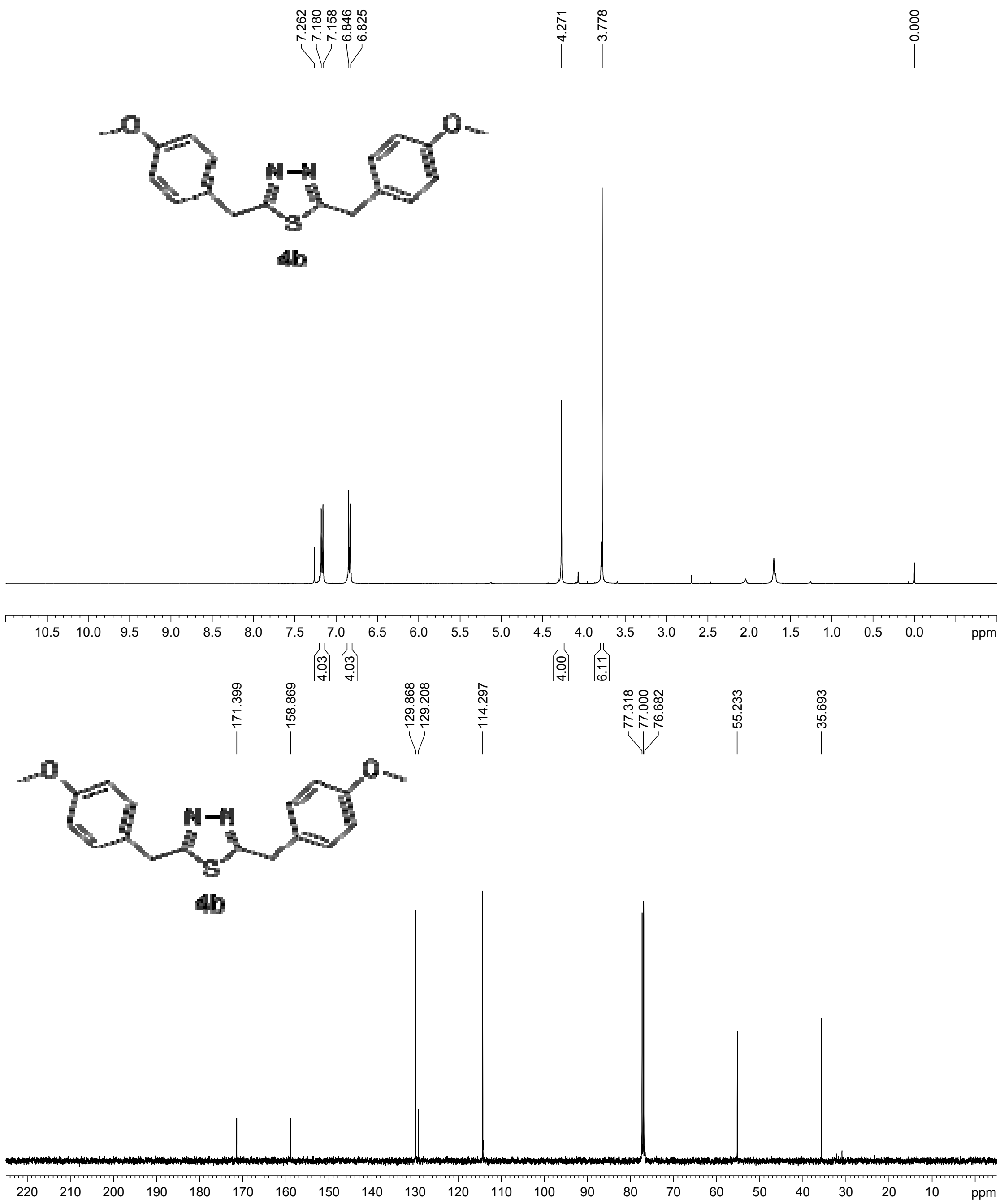

Figure S15. ^{1}H-NMR and ^{13}C-NMR of compound **4b**.

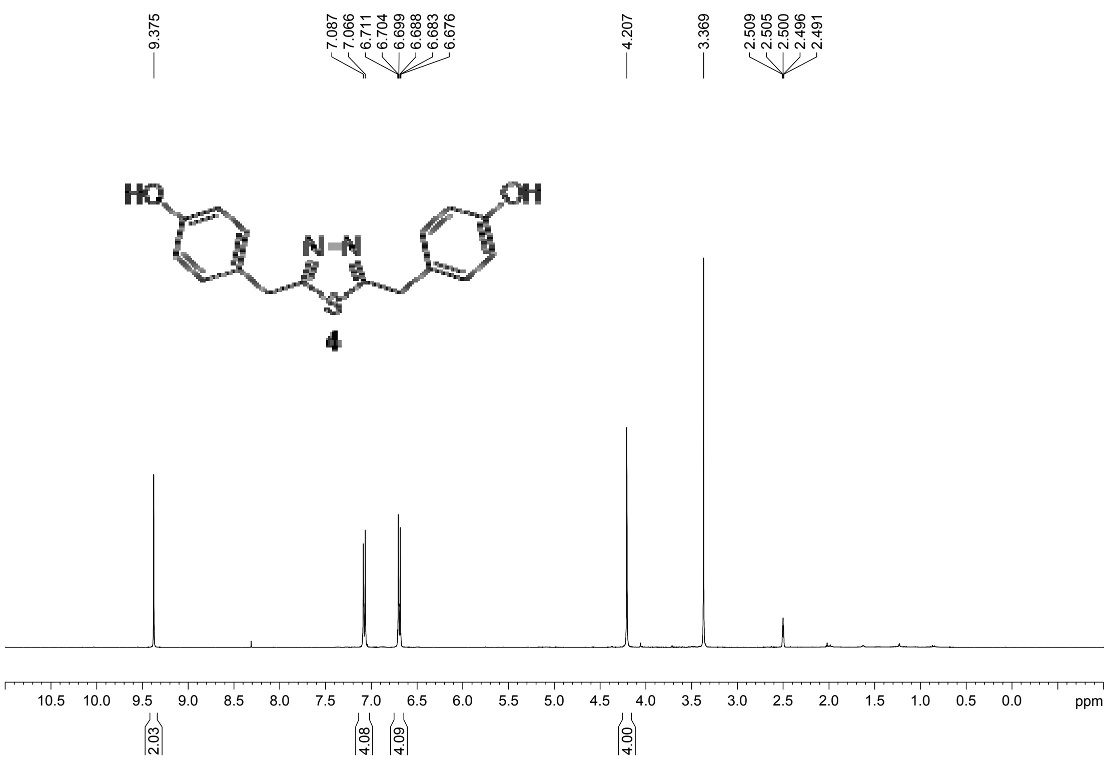

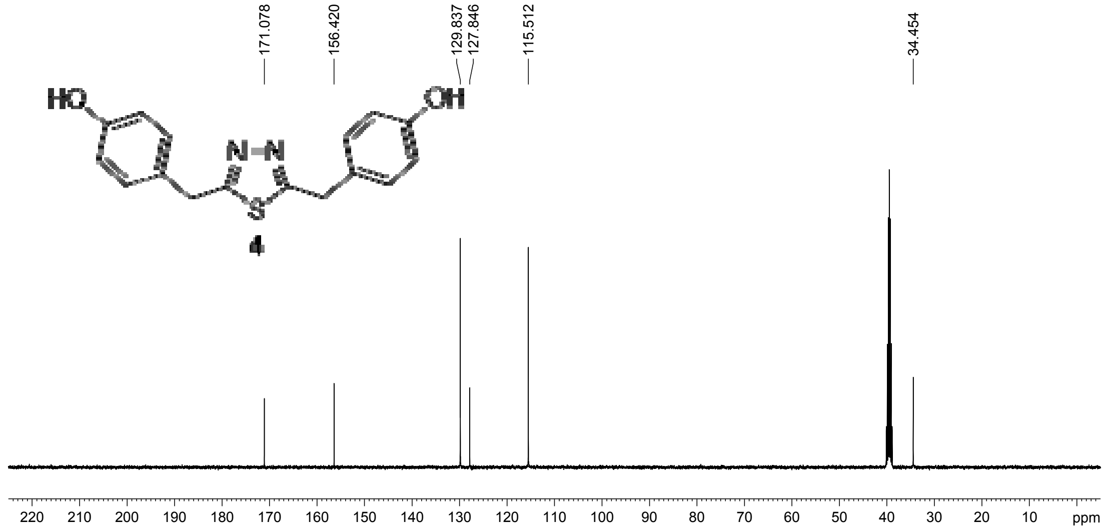

Figure S16. ^{1}H-NMR and ^{13}C-NMR of compound **4**.

2. HPLC and ^{13}C-NMR Comparing Analysis

2.1. HPLC Comparing Analysis for Compound 2

Mobile phase: CH_3CN-H_2O with a linear gradient of CH_3CN from 10% to 65% in 30 min. The flow rate of 1.0 mL/min with UV detection at 224 nm using Venusil XBP C_{18} column (5 μm, 100 Å, 4.6 × 250 mm i.d), 30 °C.

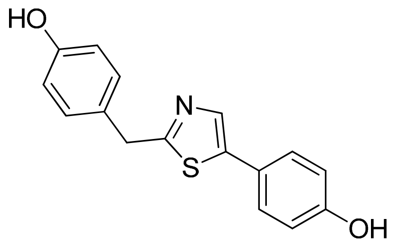

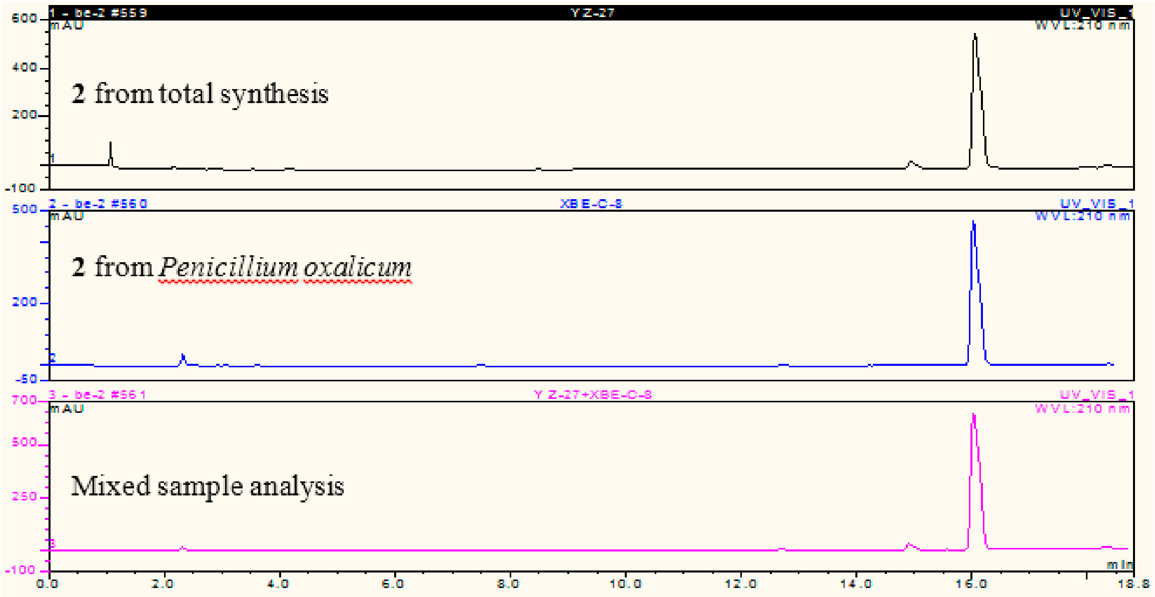

Figure S17. HPLC comparing analysis for compound **2**.

2.2. ^{13}C-NMR Comparing Analysis for Compound **1** and **2**

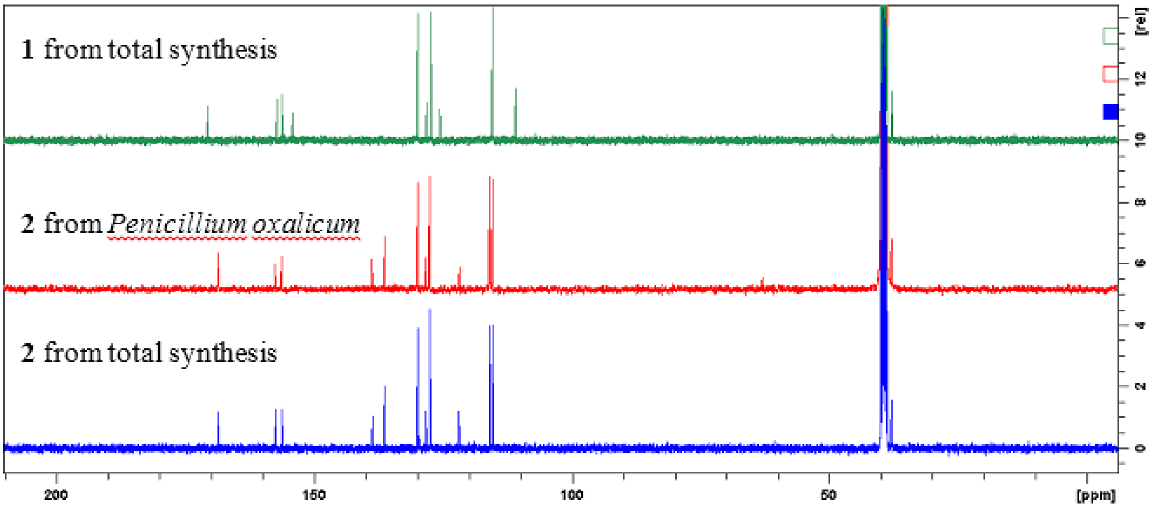

Figure S18. ^{13}C-NMR comparing analysis for compound **1** and **2**.

2.3. HPLC Comparing Analysis for Compound **3**

Mobile phase: MeCN-H_2O with a linear gradient of MeCN from 10% to 100% in 30 min. The flow rate of 1.0 mL/min with UV detection at 224 nm using Venusil XBP C_{18} column (5 μm, 100 Å, 4.6 × 250 mm i.d), 30 °C.

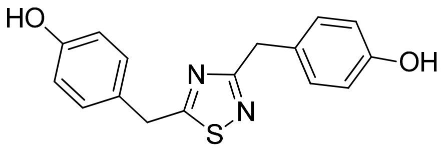

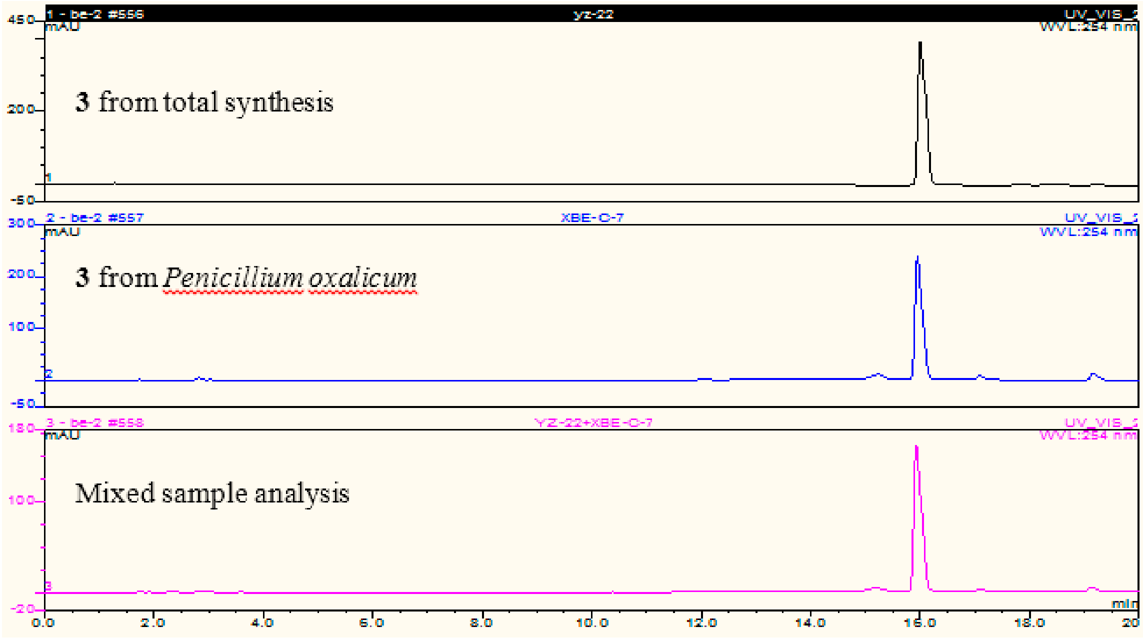

Figure S19. HPLC comparing analysis for compound **3**.

2.4. ^{13}C-NMR Comparing Analysis for Compound **3** *and* **4**

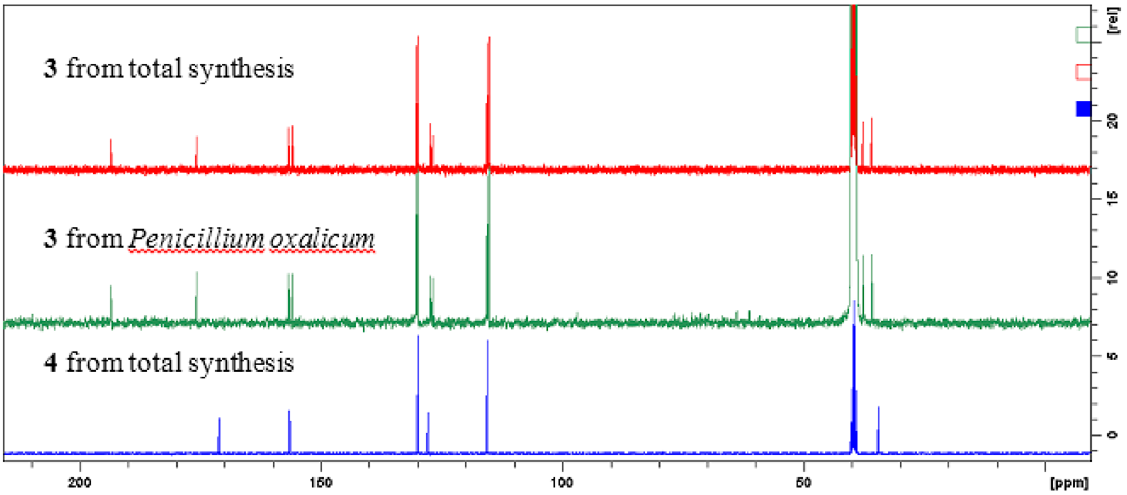

Figure S20. ^{13}C-NMR comparing analysis for compound **3** and **4**.

3. The Crystal Structure of 1g with Numbered Atoms

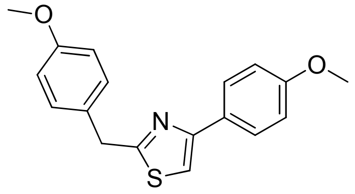

1g

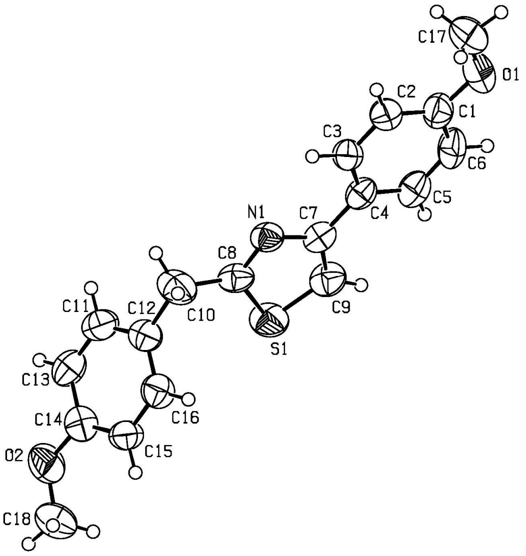

Figure S21. The crystal structure of compound **1g** with numbered atoms.

4. The Crystal Structure of 2 with Numbered Atoms

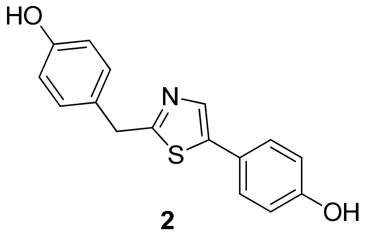

2

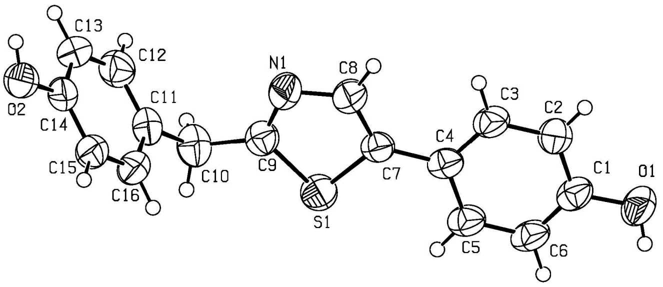

Figure S22. The crystal structure of compound **2** with numbered atoms.
